# Assessment of the relationship between serum xanthine oxidase levels and type 2 diabetes: a cross-sectional study

**DOI:** 10.1038/s41598-022-25413-w

**Published:** 2022-12-02

**Authors:** Mahmudul Hasan, Khandaker Atkia Fariha, Zitu Barman, Ananya Dutta Mou, Rakib Miah, Ahsan Habib, Humaira Rashid Tuba, Nurshad Ali

**Affiliations:** Department of Biochemistry and Molecular Biology, Shahjalal University of Science and Technology, Sylhet, 3114 Bangladesh

**Keywords:** Endocrine system and metabolic diseases, Adrenal gland diseases

## Abstract

Xanthine oxidase (XO) is an enzyme associated with purine metabolism. The relationship between XO levels and type 2 diabetes (T2D) is not clear yet or little is known so far. Therefore, we conducted a cross-sectional study to determine the association of XO levels with T2D in a Bangladeshi adult cohort. A total of 325 participants (234 males and 91 females) were enrolled in the study. The participants were divided into three groups; diabetic (n = 173), prediabetic (n = 35), and non-diabetic control (n = 117). Serum levels of XO were measured by enzyme-linked immunosorbent assay (ELISA) and other biochemical parameters including fasting blood glucose (FBG), serum uric acid (SUA), and lipid profile markers measured by colorimetric methods. Participants with T2D were confirmed according to the definition of the American Diabetic Association. The association between serum XO levels and T2D was determined by logistic regression models. The mean level of serum XO was significantly higher in females (6.0 ± 3.7 U/L) compared to male (4.0 ± 2.8 U/L) participants (p < 0.001). In contrast, males had a higher mean level of SUA (6.1 ± 1.9 mg/dL) than female (4.4 ± 1.9 mg/dL) participants (p < 0.001). The mean level of XO was significantly higher in the diabetic group (5.8 ± 3.6 U/L) compared to the prediabetic (3.7 ± 1.9 U/L) and control (2.9 ± 1.8 U/L) groups (p < 0.001). On the other hand, the mean SUA concentration was significantly lower in the diabetic group than in the other two groups (p < 0.001). A significant increasing trend was observed for FBG levels across the XO quartiles (p < 0.001). A decreasing trend was found for SUA levels in the XO quartiles (p < 0.001). Serum levels of XO and SUA showed a positive and negative correlation with FBG, respectively. In regression analysis, serum XO levels showed an independent association with T2D. In conclusion, this study reports a positive and independent association between XO levels and T2D in Bangladeshi adults. Monitoring serum levels of XO may be useful in reducing the risk of T2D. Further research is needed to determine the underlying mechanisms of the association between elevated XO levels and T2D.

## Introduction

Diabetes is a major concern in the healthcare system worldwide. About 1 in 11 adults globally are diabetic, 90% of them have type 2 diabetes mellitus (T2DM), and Asia is counted as the epicentre of the T2DM epidemic^1^. The prevalence of T2D is rapidly increasing both in developed and developing nations and has been recognized as a serious health issue due to its long-term complications and economic burden^1^. In Asia, about 80% of diabetic patients live in low- and middle-income countries, and the population in South-East Asian countries are particularly affected by diabetes^1^. The prevalence of T2D and associated complications are substantially increasing in the Bangladeshi population over the last few decades creating a huge burden on the national economy of the country.

Xanthine oxidase (XO) is a metalloflavoenzyme that catalyzes the oxidation of hypoxanthine to xanthine and xanthine to uric acid^2^. In addition to uric acid production, XO also plays an important role in generating some oxidants, which may influence oxidative damage in diabetes^3,4^. Therefore, it has been suggested that inhibition of XO may prevent oxidative stress in diabetes^3^. Some preclinical data also indicated a higher level of malondialdehyde (MDA) in plasma, glutathione redox ratio and increased hepatic protein oxidation in diabetic rats compared to non-diabetic rats^5^. These oxidative stress markers were found to be decreased following XO inhibitor treatment such as allopurinol^5^. Serum level of XO has also been suggested to be linked with cardiovascular complications^6,7^ and diabetic neuropathy in experimental models^8^. Moreover, plasma XO levels are correlated with indices of insulin resistance and liver dysfunction in patients with T2D and metabolic syndrome^9^. Elevated levels of serum uric acid (SUA) can lead to gout development and are associated with several disease conditions including obesity, diabetes, hypertension, liver and kidney dysfunction, metabolic syndrome, and cardiovascular diseases^10–15^. Some previous studies reported a positive association between SUA and T2D^16–18^ whereas other studies reported a reverse or no correlation with T2D^15,19,20^. Such inconsistency within the study findings raises doubts regarding the potential role of SUA in T2D development. In this case, an alternative biomarker of SUA such as XO would be useful which may explain its causal relationship with T2D. Recent advancements in technologies have provided facilities to quantitatively measure XO levels even present at low levels in human serum or plasma^21^. Although there are some reports on the association between XO and cardiovascular diseases^14,22^, little is known about the relationship between serum XO levels and T2DM. A previous study, conducted on diabetes-free individuals (aged > 30 years) reported an association between XO levels and the risk of developing T2DM^23^. However, further studies are needed to confirm the previous study findings in other ethnic populations. Moreover, the relationship between XO and diabetes in all age groups remains unknown or poorly understood. Furthermore, the underlying mechanisms between XO levels and the development of T2DM need to be investigated in future studies. Considering these aspects, we have conducted a cross-sectional study to assess the relationship between serum XO levels and the prevalence of T2D in Bangladeshi adults.

## Methods

### Study area and population

This cross-sectional study was conducted from October 2019 to September 2020 at the Department of Biochemistry and Molecular Biology, Shahjalal University of Science and Technology, Sylhet, Bangladesh. A total of 325 subjects (234 males and 91 females, age ≥ 20 years) were enrolled in the study of which 117 participants were non-diabetic, 35 were prediabetes and 173 were diabetic. Diabetic participants were enrolled at Sylhet Diabetic hospital who underwent there for their routine diabetes and health checks up. The other study subjects were randomly selected from Sylhet city regions. As inclusion criteria, both genders aged ≥ 20 years and participants free from severe illness were included in the study. As exclusion criteria, pregnant women, lactating mothers, individuals with a history of drug addiction and alcohol consumption and participants with anti-hyperuricemic drug intake were excluded from the study. We also excluded participants with self-reported renal and hepatic diseases, hypothyroidism, and any infectious diseases. The study protocol was approved by the internal ethics committee exists at the Department of Biochemistry and Molecular Biology, Shahjalal University of Science and Technology, Sylhet, and the ethics committee of Sylhet Diabetic Hospital, Sylhet (Reference no 02/BMB/2019). All methods of the study were carried out in accordance with relevant guidelines and regulations. Informed consent was obtained from all subjects before inclusion in the study.

### Data collection

Anthropometric data like weight, height, waist circumferences (WC) and hip circumferences (HC) were measured following a standard procedure described elsewhere^24–31^. Participants’ health status-related information such as the presence of hypertension, diabetes mellitus, and other chronic diseases were also recorded in the questionnaire form. Height was measured in centimetres (cm) using a measuring tape and weight was measured in kilograms (kg) by a digital electronic LCD weighing machine (Beurer 700, Germany). Body mass index (BMI) was calculated as body weight in kg divided by body height in meters squared (kg/m^2^). The systolic and diastolic blood pressure (SBP and DBP, respectively) were measured using digital blood pressure (BP) machine (Omron M10, Tokyo Japan).

### Fasting blood sample collection and biochemical analysis

The participants enrolled in the study were in overnight fasting condition at least for 10–12 h before providing the blood sample in the morning. Blood samples were collected from the participants in a plain dry vacutainer tube using disposable needles and syringes. The blood samples were collected by trained personnel. After collection, the blood samples were placed in the icebox until serum separation. Then blood samples were centrifuged by an ultra-centrifuge machine (Sorvall ST 8R Centrifuge, Thermo Scientific, Germany) at 4400 rpm for 10 min for separation of serum. After centrifugation, the serum samples were collected and stored at − 80 °C in the Departmental laboratory until biomarker analysis.

Serum xanthine oxidase (XO) levels were measured by enzyme-linked immunosorbent assay (ELISA) using an ELISA plate reader (ELISA reader, Apollo 11 LB 913, Berthold, Germany). The other biochemical parameters like fasting blood glucose (FBG), serum uric acid (SUA), serum creatinine, serum albumin (SA) and lipid profile (TC: total cholesterol, TG: triglyceride, HDL: high-density lipoprotein, and LDL: low-density lipoprotein) were measured by colorimetric method using commercially available kits (Human Diagnostic, Germany) according to manufacturer’s protocol with a biochemistry analyzer (Humalyzer 3000, USA). All the measurements were maintained through regular standard calibration.

### Measurement of serum XO

A commercially available ELISA kit, purchased from MyBioSource company, USA (Human XO ELISA Kit, Cat No: MBS774009) was used for the determination of XO in the serum samples. In brief, standards and serum samples were added to the antibody pre-coated micro-wells and then HRP-Conjugate solution was added to the wells to form an immune complex. After incubation at 37 °C for 1 h, the unbound enzyme was removed through washing. The substrates were added, then the solution colour turned blue, and finally changed into yellow. The colour intensity was positively correlated with the levels of XO present in the serum sample. Within 15 min, the absorbance (OD) of each well was measured at 450 nm by an ELISA reader (Apollo 11 LB 913, Berthold, Germany). Finally, XO levels in the samples were calculated based on the standard graph prepared in the excel sheet.

### Diagnostic criteria

Diabetes was defined according to the American Diabetes Association 2020 as a fasting blood plasma glucose level ≥ 126 mg/dL (7 mmol/L), non-fasting plasma glucose ≥ 200 mg/dL (11.1 mmol/L)^32^, or self-reported recent use of insulin or hypoglycemic drugs. Prediabetes was defined as a fasting plasma glucose level of 100 mg/dL to 125 mg/dL (5.6 mmol/L to 6.9 mmol/L)^32^. Non-diabetic individuals were identified based on fasting plasma glucose levels < 100 mg/dL (< 5.6 mmol/L)^32^. In the present study, hyperuricemia was defined as SUA concentration > 7.0 mg/dL (416.4 µmol/L) in men or > 6.0 mg/dL (356.9 µmol/L) in women^10,13^.

### Statistical analysis

All statistical analyses were performed using SPSS version 25.0. Baseline data are presented as mean ± standard deviation (SD) with maximum values in parentheses. The differences in baseline variables between gender and case–control groups were determined by the independent sample t-test. Pearson’s correlation coefficient was used to determine the correlation between the variables. One-way ANOVA was performed to determine differences among diabetic, prediabetic and non-diabetic groups. Differences and correlations were considered significant at p < 0.05. Association between serum XO levels and T2D was evaluated by multinomial logistic regression analysis.

## Results

### Baseline characteristics of the study subjects

**Figure 1:**
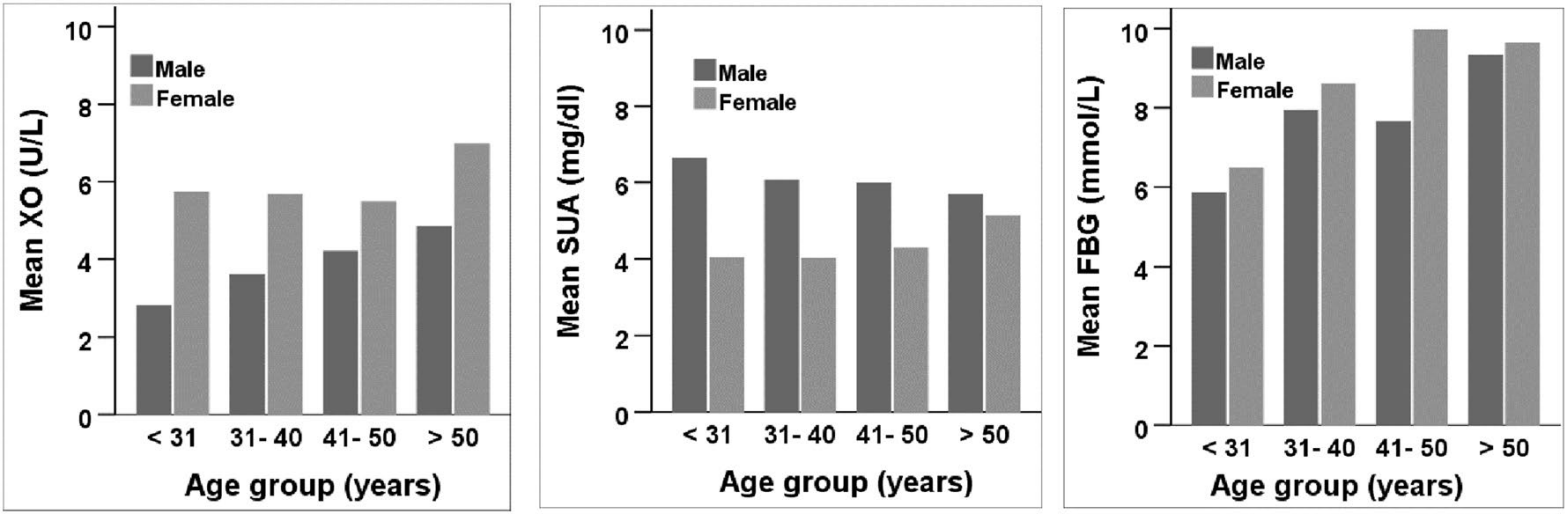
Age and sex-specific mean serum levels of FBG, XO, and SUA.

The baseline characteristics of the study subjects are presented in Table 1. Of the 325 subjects, 234 (72%) were males and 91 (28%) were females. About 46% of males and 74% of females were diabetic. The mean XO level was significantly higher in females (6.0 ± 3.7 U/L) compared to male (4.0 ± 2.8 U/L) participants (p < 0.001). In contrast, the mean level of SUA was significantly higher in males (6.1 ± 1.9 mg/dL) than in female (4.4 ± 1.9 mg/dL) participants (p < 0.001). Other parameters like serum level of creatinine, SA and eGFR were higher in males, whereas, mean age, BMI, FBG, XO, TC, and LDL were higher in females. The participants were also categorized into four different age groups (Fig. 1). An increasing trend was observed for FBG and XO with increasing age in the groups. However, SUA showed a decreasing trend especially in males with increasing age in the groups.

**Table 1:** Gender-wise baseline characteristics of the study subjects.

Variables	Total	Male	Female	p-value
Number, n (%)	325	234 (72%)	91 (28%)	–
Age (years)	43.6 ± 12.7 (85.0)	43.1 ± 12.6 (85.0)	44.8 ± 12.7 (71.0)	0.260
Weight (kg)	65.2 ± 11.6 (105.0)	67.9 ± 10.6 (105.0)	58.2 ± 11.0 (90.0)	0.000
Height (cm)	92.8 ± 78.9 (181.0)	97.0 ± 80.2 (181.0)	81.9 ± 75.0 (160.0)	0.112
BMI (kg/m^2^)	25.2 ± 3.7 (40.0)	25.0 ± 3.4 (39)	25.7 ± 4.4 (40.0)	0.175
WC (cm)	86.6 ± 10.3 (116.0)	87.6 ± 10.0 (116.0)	84.3 ± 10.5 (113.0)	0.023
HC (cm)	91.7 ± 8.1 (113.0)	92.0 ± 8.0 (113.0)	90.8 ± 8.3 (113.0)	0.290
SBP (mmHg)	129.9 ± 17.7 (216.0)	129.8 ± 17.5 (216.0)	130.1 ± 18.4 (190.0)	0.906
DBP (mmHg)	84.3 ± 9.7 (118.0)	84.1 ± 9.9 (118.0)	84.6 ± 9.3 (112.0)	0.667
PP (Beats/min)	79.5 ± 12.0 (124.0)	78.1 ± 12.1 (122.0)	82.7 ± 11.1 (124.0)	0.004
FBG (mmol/L)	8.1 ± 4.4 (26.9)	7.8 ± 4.4 (26.9)	9.0 ± 4.2 (26.4)	0.024
XO (U/L)	4.5 ± 3.2 (18.9)	4.0 ± 2.8 (17.4)	6.0 ± 3.7 (18.9)	0.000
SUA (mg/dL)	5.6 ± 2.0 (12.9)	6.1 ± 1.9 (12.9)	4.4 ± 1.9 (9.9)	0.000
Creatinine (mg/dL)	0.9 ± 0.3 (2.5)	0.9 ± 0.3 (2.5)	0.7 ± 0.3 (2.3)	0.000
eGFR (mL/min/1.73 m^2^)	89.5 ± 18.8 (149.1)	90.1 ± 18.9 (147.0)	87.6 ± 18.6 (149.1)	0.371
SA(g/L)	45.3 ± 11.1 (94.4)	46.8 ± 11.2 (94.4)	41.1 ± 9.8 (68.4)	0.007
TG (mg/dL)	202.7 ± 123.2 (812.6)	202.2 ± 119.7 (726.6)	203.8 ± 132.4 (812.6)	0.920
TC (mg/dL)	219.5 ± 87.7 (584.0)	213.0 ± 81.6 (561.9)	236.5 ± 100.2 (584.0)	0.041
HDL (mg/dL)	34.6 ± 13.4 (112.4)	34.0 ± 14.2 (112.4)	36.4 ± 10.9 (67.8)	0.172
LDL (mg/dL)	145.6 ± 81.9 (517.2)	139.5 ± 72.3 (514.9)	161.3 ± 101.3 (517.2)	0.079
Diabetes (%)	53.2	45.3	73.6	–

Values are presented as mean ± SD with maximum values in parentheses.

p-values are given for differences between the gender groups and obtained from an independent sample t-test. T2D was defined as FBG ≥ 7 mmol/L in participants.

### Baseline characteristics in control, prediabetic, and diabetic subjects

**Figure 2:**
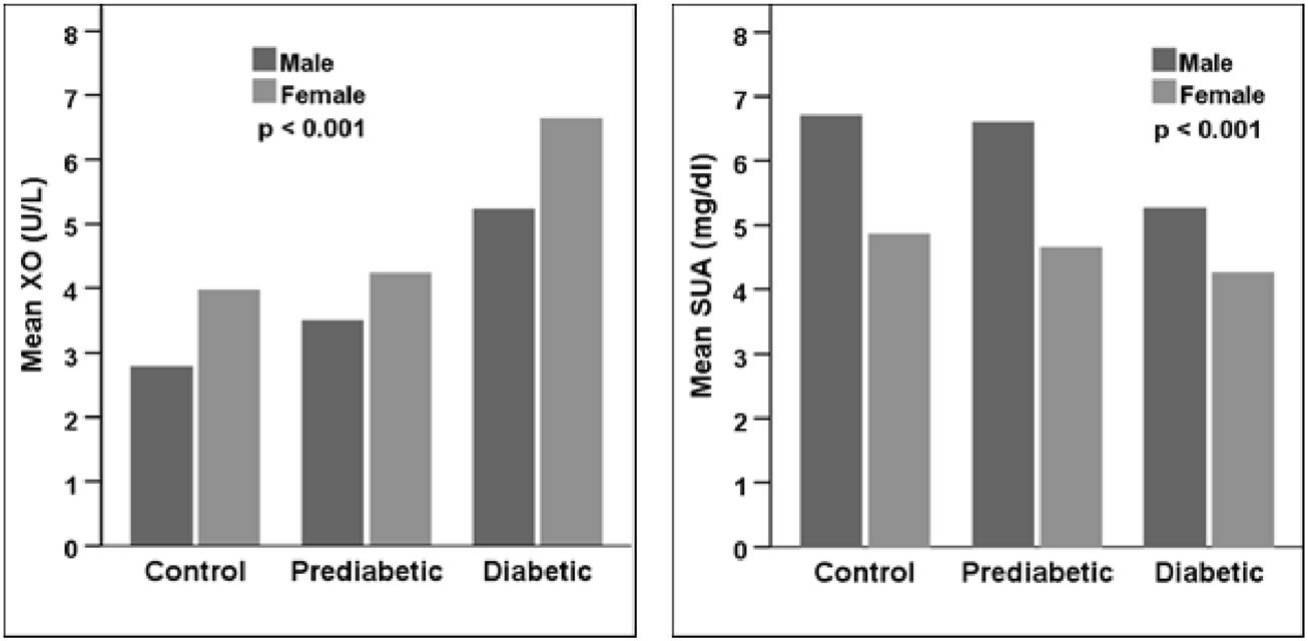
The mean of level XO and SUA in control, prediabetic, and diabetic groups. p-values are given for the mean differences of XO and SUA within the groups. p-values were derived from one-way ANOVA.

Table 2 shows the participant’s baseline characteristics in nondiabetic, prediabetic, and diabetic groups. Of total 325 subjects 117 (36%) were in control, 35 (10.77%) were prediabetic and 173 (53.23%) were diabetic groups. The mean XO level was significantly higher in the diabetic group (5.8 ± 3.6 U/L), compared to the prediabetic (3.7 ± 1.9 U/L) and control (2.9 ± 1.8 U/L) groups (p < 0.001) (Table 2, Fig. 2). On the other hand, SUA was significantly lower in the diabetic group (4.9 ± 1.9 mg/dL) than in the control (6.5 ± 1.8 mg/dL) and prediabetic (6.1 ± 1.7 mg/dL) groups (p < 0.001) (Table 2, Fig. 2). The other variables like mean age, BMI, SBP, FBG, TG, TC and HDL were higher in the diabetic group than in the control and prediabetic groups (p < 0.05 at least for all cases). Whereas, SA and eGFR were lower in the diabetic group than in the other groups (p < 0.05 at least for both cases).

**Table 2:** Baseline characteristics in control, prediabetic, and diabetic groups.

Variables	Control	Prediabetic	Diabetic	*p*-value
Number, n (%)	117 (36%)	35 (10.77%)	173 (53.23%)	–
Age (years)	38.2 ± 10.7 (65.0)	36.0 ± 12.1 (60.0)	48.7 ± 11.7 (85.0)	0.000
Weight (kg)	66.8 ± 11.1 (98.0)	65.8 ± 9.8 (93.5)	63.9 ± 12.1 (105.0)	0.112
Height (cm)	80.1 ± 81.8 (181.0)	106.5 ± 77.1 (176.0)	98.4 ± 76.6 (178.0)	0.085
BMI (kg/m^2^)	24.6 ± 3.5 (33.0)	24.9 ± 3.0 (31.0)	25.6 ± 4.0 (40.0)	0.049
WC (cm)	85.1 ± 10.4 (110.0)	86.0 ± 9.2 (105.0)	87.1 ± 10.3 (116.0)	0.438
HC (cm)	92.2 ± 8.3 (113.0)	93.7 ± 6.1 (108.0)	91.1 ± 8.2 (113.0)	0.302
SBP (mmHg)	123.0 ± 13.9 (167.0)	130.8 ± 15.8 (160.0)	134.3 ± 18.9 (216.0)	0.000
DBP (mmHg)	82.6 ± 10.8 (118.0)	85.4 ± 11.2 (112.0)	85.2 ± 8.5 (115.0)	0.066
PP (Beats/min)	75.8 ± 11.9 (122.0)	79.6 ± 10.9 (106.0)	83.3 ± 11.2 (124.0)	0.000
FBG (mmol/L)	4.8 ± 0.4 (5.5)	6.0 ± 0.4 (6.8)	10.8 ± 4.5 (26.9)	0.000
XO (U/L)	2.9 ± 1.8 (8.2)	3.7 ± 1.9 (7.8)	5.8 ± 3.6 (18.9)	0.000
SUA (mg/dL)	6.5 ± 1.8 (12.9)	6.1 ± 1.7 (9.6)	4.9 ± 1.9 (10.6)	0.000
Creatinine (mg/dL)	0.9 ± 0.2 (1.3)	0.8 ± 0.2 (1.2)	0.9 ± 0.4 (2.5)	0.631
eGFR (mL/min/1.73 m^2^)	92.8 ± 16.7 (149.1)	91.1 ± 12.1 (114.2)	86.0 ± 21.4 (147.0)	0.028
SA (g/L)	50.4 ± 14.4 (94.4)	48.4 ± 8.0 (68.4)	42.6 ± 9.2 (67.0)	0.001
TG (mg/dL)	178.2 ± 96.6 (516.4)	183.2 ± 113.5 (474.4)	225.3 ± 138.6 (812.6)	0.006
TC (mg/dL)	204.3 ± 58.1 (426.1)	216.4 ± 67.5 (377.7)	231.9 ± 106.7 (584.0)	0.041
HDL (mg/dL)	32.6 ± 10.9 (82.0)	32.6 ± 8.8 (55.0)	36.6 ± 15.5 (112.4)	0.044
LDL (mg/dL)	136.2 ± 52.9 (317.2)	149.1 ± 57.4 (307.5)	152.1 ± 101.7 (517.2)	0.290

Values are presented as mean ± SD with maximum values in parentheses.

p-values are given for differences in control, prediabetic, and diabetic groups. p-values are obtained from one-way ANOVA.

### Characteristics of the participants according to XO quartiles

**Figure 3:**
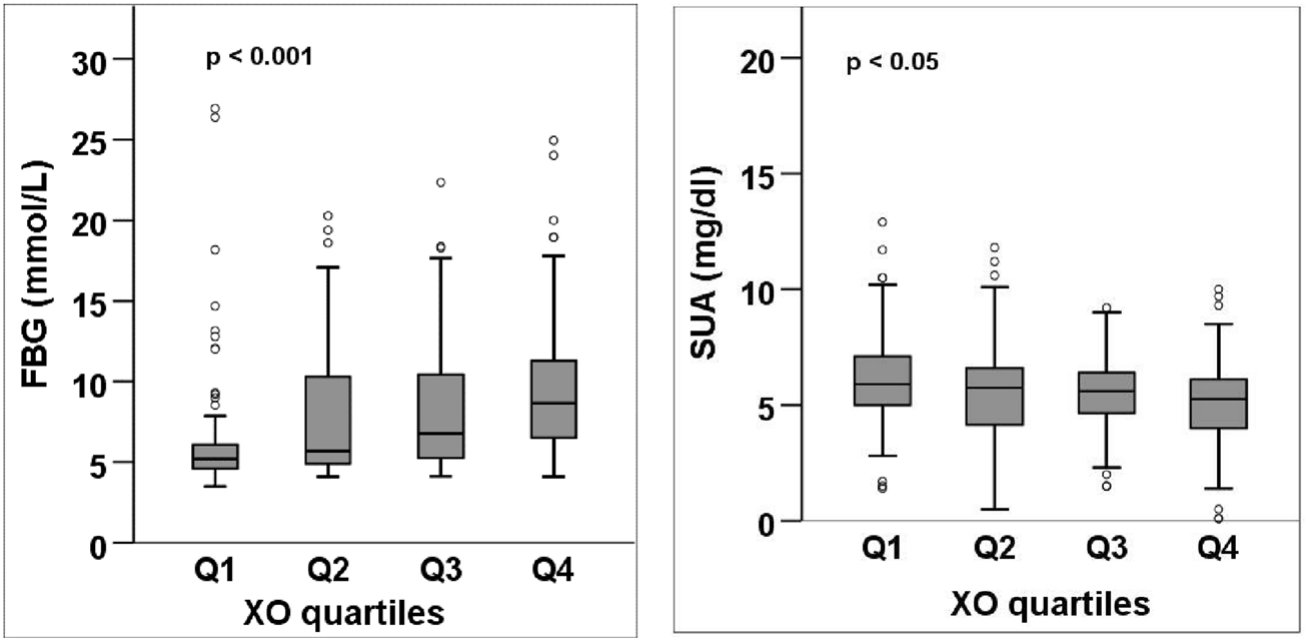
FBG and SUA levels across the XO quartiles. p-values are obtained from one-way ANOVA.

Serum levels of XO were divided into four quartiles: Q1 (< 2.23 U/L), Q2 (2.23–3.72 U/L), Q3 (3.73–6.14 U/L), and Q4 (> 6.14 U/L). A significant increasing trend was observed for mean FBG across the XO quartiles (p < 0.001) (Table 3, Fig. 3). An increasing trend was also observed for the mean level of SBP, and creatinine in the XO quartiles (at least p < 0.05 for all cases). In contrast, a significant decreasing trend was found for the mean level of SUA, SA and eGFR across the XO quartiles (p < 0.05) (Table 3, Fig. 3).

**Table 3:** Baseline characteristics of the study subjects in the XO quartiles.

Variables	XO (U/L)				*p*-value
	Q1 (< 2.23)	Q2 (2.23–3.72)	Q3 (3.73–6.14)	Q4 (> 6.14)	
Number (n)	81	81	82	81	–
Age (years)	39.1 ± 12.1 (71.0)	42.7 ± 12.7 (70.0)	46.1 ± 12.7 (85.0)	46.4 ± 12.0 (70.0)	0.000
Weight (kg)	67.9 ± 9.1 (92.0)	66.1 ± 10.6 (96.6)	65.9 ± 13.8 (105.0)	60.7 ± 11.0 (98.0)	0.000
Height (cm)	76.7 ± 81.7 (181.0)	107.6 ± 75.7 (178.0)	105.0 ± 77.2 (177.0)	81.4 ± 77.7 (175.0)	0.019
BMI (kg/m^2^)	25.2 ± 3.5 (38.0)	25.3 ± 3.6 (34.0)	25.3 ± 4.0 (40.0)	24.9 ± 3.9 (35.0)	0.901
WC (cm)	87.9 ± 8.9 (104.1)	88.6 ± 11.5 (116.0)	87.5 ± 10.0 (111.0)	83.3 ± 9.6 (111.0)	0.013
HC (cm)	94.1 ± 7.3 (108.0)	93.0 ± 9.1 (113.0)	91.5 ± 8.1 (110.0)	89.3 ± 7.1 (113.0)	0.008
SBP (mmHg)	121.6 ± 12.7 (160.0)	128.0 ± 16.5 (180.0)	134.6 ± 17.9 (216.0)	135.2 ± 19.7 (190.0)	0.000
DBP (mmHg)	82.8 ± 9.0 (112.0)	83.0 ± 9.6 (110.0)	85.3 ± 9.8 (110.0)	85.9 ± 10.3 (118.0)	0.097
PP (Beats/min)	75.4 ± 9.3 (96.0)	80.7 ± 12.3 (122.0)	81.4 ± 14.6 (124.0)	81.2 ± 10.9 (112.0)	0.008
FBG (mmol/L)	6.6 ± 4.1 (26.9)	7.9 ± 4.3 (20.3)	8.5 ± 4.2 (22.3)	9.6 ± 4.4 (24.9)	0.000
XO (U/L)	1.5 ± 0.6 (2.2)	2.9 ± 0.4 (3.7)	4.7 ± 0.7 (5.9)	9.0 ± 2.9 (18.9)	0.000
SUA (mg/dL)	6.1 ± 2.0 (12.9)	5.6 ± 2.2 (11.8)	5.5 ± 1.6 (9.2)	5.1 ± 2.0 (10.0)	0.022
Creatinine (mg/dL)	0.9 ± 0.4 (2.5)	0.9 ± 0.2 (1.5)	0.9 ± 0.3 (2.0)	0.8 ± 0.3 (2.1)	0.030
eGFR (mL/min/1.73 m^2^)	93.7 ± 19.2 (149.1)	92.5 ± 15.4 (126.2)	87.2 ± 21.2 (123.1)	84.2 ± 17.1 (116.8)	0.019
SA (g/L)	53.0 ± 14.1 (94.4)	44.7 ± 9.6 (70.6)	43.0 ± 7.7 (60.6)	40.7 ± 8.9 (54.4)	0.000
TG (mg/dL)	194.3 ± 121.9 (675.2)	182.8 ± 118.1 (583.3)	211.7 ± 126.7 (726.6)	219.4 ± 124.4 (812.6)	0.284
TC (mg/dL)	204.9 ± 66.3 (463.5)	237.2 ± 108.5 (562.8)	218.4 ± 89.6 (584.0)	219.2 ± 81.7 (469.8)	0.190
HDL (mg/dL)	31.4 ± 9.2 (63.2)	34.9 ± 13.2 (91.0)	36.5 ± 16.7 (112.4)	35.6 ± 12.9 (82.0)	0.112
LDL (mg/dL)	135.5 ± 63.5 (324.6)	167.6 ± 101.0 (517.2)	139.8 ± 79.4 (506.5)	141.5 ± 79.1 (368.3)	0.087

Values are presented as mean ± SD with maximum values in parentheses.

p-values are obtained from one-way ANOVA.

### Correlation of XO with FBG, and SUA

**Figure 4:**
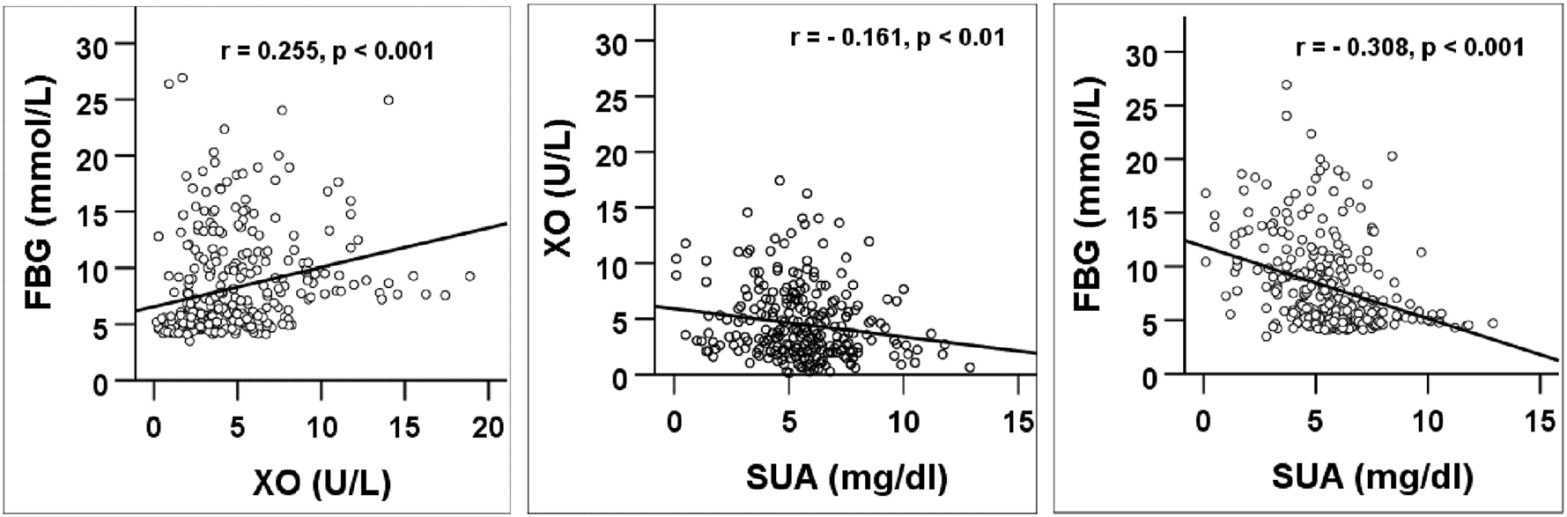
Correlation of XO with FBG and SUA and SUA with FBG. p values are obtained from the Pearson correlation coefficient test.

Figure 4 shows the correlation of XO levels with FBG and SUA. A significant positive correlation was observed between serum XO levels and FBG (p < 0.001). However, a significant negative correlation was found between SUA and FBG (p < 0.001). The SUA also showed a negative correlation with XO (p < 0.01).

### Association between serum XO and T2D

In logistic regression analysis, three models were applied to evaluate the association between XO levels and T2D (Table 4). In model 1, age, BMI, SBP and DBP were adjusted. Model 2 was adjusted for variables included in model 1 and SUA. Finally, model 3 was adjusted for variables included in models 1 and 2 and additionally lipid profile markers. In all models, XO showed a significant and independent association with T2D (p < 0.001). The strength of this significance was higher in male than in female participants.

**Table 4:** Evaluation of the association between XO and T2D.

	B	SE	Wald	Df	OR (95% Cl)	p-value
**Male**						
Model 1	0.334	0.085	15.479	1	1.396 (1.182–1.649)	0.000
Model 2	0.350	0.093	14.196	1	1.419 (1.183–1.702)	0.000
Model 3	0.468	0.133	12.403	1	1.597 (1.231–2.071)	0.000
**Female**						
Model 1	0.342	0.149	5.265	1	1.408 (1.051–1.887)	0.022
Model 2	0.333	0.151	4.840	1	1.395 (1.037–1.878)	0.028
Model 3	0.700	0.310	5.095	1	2.014 (1.097–3.699)	0.024
**Overall**						
Model 1	0.383	0.073	27.830	1	1.467 (1.272–1.692)	0.000
Model 2	0.354	0.076	21.543	1	1.424 (1.227–1.654)	0.000
Model 3	0.472	0.105	20.172	1	1.603 (1.304–1.969)	0.000

Association between XO levels and T2D was determined by Multinomial logistic regression. The dependent variable was T2D (yes) and the independent variable was XO (U/L). The reference category is control (non-T2D).

Model 1: adjusted for age, BMI, SBP and DBP.

Model 2: model 1 + SUA.

Model 3: model 2 + lipid markers (TG, TC, HDL, LDL), SA, eGFR and smoking status.

*OR* odds ratio, *CI* confidence interval, *SE* standard error.

## Discussion

This study examined the association between elevated serum XO levels and T2D in Bangladeshi adults. Up to now, there is limited information on the association between XO and T2D. Therefore, our study findings will add some important information that may be useful in future research on this topic.

In our study, the mean XO level was significantly higher in the diabetic group compared to the prediabetic and control group, whereas the opposite finding was observed for SUA within the groups. An increasing trend for FBG and a decreasing trend for SUA were found in the XO quartiles. In the sex groups, the mean level of XO was significantly higher in females compared to males. In contrast, the mean level of SUA was significantly higher in males than in females.

We also observed a higher mean XO level in the aged (> 50 years) population group. An increasing trend in the mean XO level was found with increasing age, especially in males. In contrast, a decreasing trend in the mean level of SUA was found with increasing age in male subjects. The incidence of diabetes is higher with increased age^33,34^ which may be related to insufficient or lack of insulin secretion following poor pancreatic function in aged people^35^. In animal studies, a positive correlation was found between XO levels and age in rats^36^. The elevated XO levels in plasma in aged mice are in agreement with studies conducted in rats and humans, where a positive correlation was found between XO levels and age^37^. Regarding SUA, a higher mean level of SUA was found in Chinese-aged people^38^, however, in another study in Korea, SUA levels did not increase in the aged people^39^. Several studies suggest that SUA is more dominant in the younger age groups and decreases during ageing^40–43^.

In the present study, a significant positive correlation was found between serum XO levels and FBG, whereas a significant negative correlation was found for SUA with XO and FBG. Therefore, SUA is possibly not an independent risk factor for T2D. XO and SUA are closely correlated in the metabolic process, as SUA is the final oxidation product of purine catabolism and XO mediates the catalyzing steps of xanthine to uric acid^2^. Another study showed that XO was increased according to elevated FBG in Chinese, Malay, and Indian people^4^. Consistent with our findings, a negative correlation was also found between SUA and FBG in several previous studies^15,20,44^.

However, a study reported no significant correlation between SUA and FBG in diabetic individuals in India^19^. Such inconsistent findings between studies raise doubts regarding the potential role of SUA in T2D development. Some studies demonstrated that a genetic score for uric acid levels in the blood, derived from several genetic markers which do not influence the risk of T2D, suggesting that even though there is an association between SUA levels and T2D, the results are likely confounded and thus not a causal relationship^45,46^.

In regression analysis, a significant and independent association was found between XO and the prevalence of T2D in both genders. Our results are consistent with a previous study where elevated serum XO levels were independently associated with the increased risk of developing in both genders after adjustment for several confounders including SUA^23^. Another study reported a link between serum XO levels and hyperglycemia and insulin resistance^4^. XO levels have also been found to be correlated with indices of both insulin resistance and liver dysfunction in patients with T2D and metabolic syndrome^9^. In cross-sectional studies, it has been suggested that XO level is associated with cardiometabolic risk factors both in children and adults^47,48^. A study reported that the positive effects of XO inhibitors on chronic kidney disease related to increased cardiovascular risk are due to reduced oxidative stress, independent of SUA concentrations^6^. Some studies showed the effectiveness of allopurinol, an inhibitor of XO, to prevent impaired relaxation and cardiac ischemia in an experimental model of insulin resistance^49^, and generalized endothelial function in T2D subjects with mild hypertension^50^, reduced oxidative injury and ameliorating cardiovascular functions in diabetic patients^51^. Other studies also observed that XO inhibitors may reduce cardiometabolic risk^52–54^. So, the relationship between XO levels and T2D suggests that controlling XO levels may reduce the risk of T2D. Future studies may confirm the utility of serum XO levels as an effective predictor for T2D.

The underlying mechanisms between XO and T2D are not well established yet. However, some proposed pathways may explain the association between XO levels and T2D. Elevated XO-mediated increased oxidative stress may be an important link to T2D, as it also generates some oxidants^55^. Oxidative stress has been suggested as a well-established pathway in the pathogenesis of diabetic complications^56^. In the purine catabolism process, XO produces high quantities of reactive oxygen species (ROS) which can enter the cell membranes and cause damage to β-cells of the pancreas which may lead to the initiation of T2D^57,58^. Moreover, it has been suggested that ROS may play key roles in the progression of insulin resistance and pancreatic β-cell dysfunction and lead to diabetes^59^. However, further experimental and population-based studies are needed to address the association between XO levels and T2D mediated by oxidative stress.

There were some limitations to our study. First, the cross-sectional nature of our data may affect the causal relationship between serum XO levels and T2D. Second, our sample size was relatively small which may not represent the entire number of T2D individuals in Bangladesh. Further studies with a large sample size would be useful to confirm our findings. Third, the findings of our study may not be applied to other ethnic populations. Fourthly, we did not have data on HbA1C, insulin resistance and urinary uric acid excretion rate which might be important confounders of diabetes and XO levels. Fifth, we did not have information on drugs used for lowering uric acid levels. Finally, we could not collect detailed information on dietary habits and alcohol consumption which may also affect the XO and SUA concentration. Despite some limitations, the major strength of the present study is that most of the risk factors of diabetes were included in the regression models to evaluate the relationship between XO and T2D. Therefore, this study findings may be used as a worthy reference in future investigations.

## Conclusions

In the present study, a significant increasing trend was observed for FBG levels across the XO quartiles. A positive and independent association was found between serum XO levels and T2D. Moreover, this study findings indicate that serum XO may be a better marker than SUA that is potentially associated with the risk of the T2D development process. Through the measurement of serum XO activities, individuals having normal levels of SUA with higher XO levels can be screened, thereby providing useful information regarding the hidden risks for T2D and related vascular diseases. Future studies are needed to determine the mechanism by which blood glucose controls ROS homeostasis and the role of XO in individuals with and without T2D. Understanding these mechanisms is of interest as they could assist in defining the pathophysiological process of cardiovascular disease in individuals with T2D and lead to the development of appropriate therapeutic approaches.

## Data Availability

The datasets used and/or analysed during the current study available from the corresponding author on reasonable request.
